# Supplementary material for: Influence of Lymphangio vascular (V) and perineural (N) invasion on survival of patients with resected esophageal squamous cell carcinoma (ESCC): a single-center retrospective study
Source: PeerJ. 2022 Mar 2;10:e12974. doi: 10.7717/peerj.12974 (PMC8898008; doi:10.7717/peerj.12974)
Supplement: Supplemental Information 2 [file peerj-10-12974-s002.docx]

1、Univariate Cox regression model analysis can be used to distinguish whether the differences were statistically significant.

**Age**

| **Variables in the Equation** | | | | | | | | |
| --- | --- | --- | --- | --- | --- | --- | --- | --- |
|  | B | SE | Wald | df | Sig. | Exp(B) | 95.0% CI for Exp(B) | |
|  |  |  |  |  |  |  | Lower | Upper |
| Age | .152 | .192 | .628 | 1 | .428 | 1.164 | .799 | 1.697 |

**Sex**

|  | | | | | | | | |
| --- | --- | --- | --- | --- | --- | --- | --- | --- |
|  | B | SE | Wald | df | Sig. | Exp(B) | 95.0% CI for Exp(B) | |
|  |  |  |  |  |  |  | Lower | Upper |
| Sex | -.484 | .231 | 4.380 | 1 | .036 | .616 | .392 | .970 |

**Tumor location**

|  | | | | | | | | |
| --- | --- | --- | --- | --- | --- | --- | --- | --- |
|  | B | SE | Wald | df | Sig. | Exp(B) | 95.0% CI for Exp(B) | |
|  |  |  |  |  |  |  | Lower | Upper |
| location |  |  | 1.279 | 2 | .528 |  |  |  |
| Lower | .076 | .291 | .068 | 1 | .794 | 1.079 | .610 | 1.909 |
| Middle | -.158 | .279 | .321 | 1 | .571 | .854 | .494 | 1.476 |

**Grade**

|  | | | | | | | | |
| --- | --- | --- | --- | --- | --- | --- | --- | --- |
|  | B | SE | Wald | df | Sig. | Exp(B) | 95.0% CI for Exp(B) | |
|  |  |  |  |  |  |  | Lower | Upper |
| Grade |  |  | .915 | 2 | .633 |  |  |  |
| well | .066 | .298 | .048 | 1 | .826 | 1.068 | .595 | 1.916 |
| moderate | -.147 | .247 | .355 | 1 | .551 | .863 | .532 | 1.400 |

**T stage**

|  | | | | | | | | |
| --- | --- | --- | --- | --- | --- | --- | --- | --- |
|  | B | SE | Wald | df | Sig. | Exp(B) | 95.0% CI for Exp(B) | |
|  |  |  |  |  |  |  | Lower | Upper |
| T | .385 | .205 | 3.515 | 1 | .061 | 1.469 | .983 | 2.197 |

| **Stage** | | | | | | | | |
| --- | --- | --- | --- | --- | --- | --- | --- | --- |
|  | B | SE | Wald | df | Sig. | Exp(B) | 95.0% CI for Exp(B) | |
|  |  |  |  |  |  |  | Lower | Upper |
| Stage III-IV | .774 | .198 | 15.219 | 1 | .000 | 2.169 | 1.470 | 3.201 |

**Primary tumor length**

|  | | | | | | | | |
| --- | --- | --- | --- | --- | --- | --- | --- | --- |
|  | B | SE | Wald | df | Sig. | Exp(B) | 95.0% CI for Exp(B) | |
|  |  |  |  |  |  |  | Lower | Upper |
| length | .252 | .191 | 1.746 | 1 | .186 | 1.287 | .885 | 1.871 |

**Tumor types**

|  | | | | | | | | |
| --- | --- | --- | --- | --- | --- | --- | --- | --- |
|  | B | SE | Wald | df | Sig. | Exp(B) | 95.0% CI for Exp(B) | |
|  |  |  |  |  |  |  | Lower | Upper |
| Tumor types |  |  | 3.621 | 2 | .164 |  |  |  |
| Ulcerative | .414 | .236 | 3.076 | 1 | .079 | 1.513 | .953 | 2.403 |
| Medullary | .097 | .249 | .151 | 1 | .698 | 1.101 | .676 | 1.794 |

**Lymphatic invasion**

|  | | | | | | | | |
| --- | --- | --- | --- | --- | --- | --- | --- | --- |
|  | B | SE | Wald | df | Sig. | Exp(B) | 95.0% CI for Exp(B) | |
|  |  |  |  |  |  |  | Lower | Upper |
| Lymphatic invasion | .936 | .205 | 20.838 | 1 | .000 | 2.551 | 1.706 | 3.813 |

Perineural invasion

|  | | | | | | | | |
| --- | --- | --- | --- | --- | --- | --- | --- | --- |
|  | B | SE | Wald | df | Sig. | Exp(B) | 95.0% CI for Exp(B) | |
|  |  |  |  |  |  |  | Lower | Upper |
| PNI | .570 | .218 | 6.817 | 1 | .009 | 1.768 | 1.153 | 2.711 |

Lymphangio vascular invasion

|  | | | | | | | | |
| --- | --- | --- | --- | --- | --- | --- | --- | --- |
|  | B | SE | Wald | df | Sig. | Exp(B) | 95.0% CI for Exp(B) | |
|  |  |  |  |  |  |  | Lower | Upper |
| LVI | .992 | .201 | 24.399 | 1 | .000 | 2.696 | 1.819 | 3.996 |

Postoperative chemotherapy

|  | B | SE | Wald | df | Sig. | Exp(B) | 95.0% CI for Exp(B) | |
| --- | --- | --- | --- | --- | --- | --- | --- | --- |
|  |  |  |  |  |  |  | Lower | Upper |
| Post-chemotherapy | -.079 | .199 | .157 | 1 | .692 | .924 | .626 | 1.364 |

**2、Multivariate Cox regression model analysis was used to distinguish whether the differences were statistically significant according to the above meaningful variables.**

|  | | | | | | | | |
| --- | --- | --- | --- | --- | --- | --- | --- | --- |
|  | B | SE | Wald | df | Sig. | Exp(B) | 95.0% CI for Exp(B) | |
|  |  |  |  |  |  |  | Lower | Upper |
| sex | -.360 | .239 | 2.272 | 1 | .132 | .698 | .437 | 1.114 |
| Ly | .901 | .429 | 4.417 | 1 | .036 | 2.461 | 1.063 | 5.701 |
| stage | -.419 | .399 | 1.104 | 1 | .293 | .658 | .301 | 1.437 |
| PNI | .147 | .236 | .390 | 1 | .532 | 1.159 | .730 | 1.838 |
| LVI | .586 | .271 | 4.682 | 1 | .030 | 1.796 | 1.057 | 3.053 |

**3、Comparison of Kaplan–Meier curves for the prognosis of patients was determined in specific situation compared in subgroups (pathological stage, status of lymphatic invasion and pathological depth of tumor invasion.)**

For I-II stage ESCC patients

| **Overall Comparisons** | | | |
| --- | --- | --- | --- |
|  | Chi-Square | df | Sig. |
| Log Rank (Mantel-Cox) | 13.749 | 1 | .000 |
| Breslow (Generalized Wilcoxon) | 16.377 | 1 | .000 |
| Tarone-Ware | 15.544 | 1 | .000 |
| The vector of trend weight is - 3, - 1, 1, 3. This is the default. | | | |

For III-IV stage ESCC patients

| **Overall Comparisons** | | | |
| --- | --- | --- | --- |
|  | Chi-Square | df | Sig. |
| Log Rank (Mantel-Cox) | 1.617 | 1 | .203 |
| Breslow (Generalized Wilcoxon) | 1.703 | 1 | .192 |
| Tarone-Ware | 1.727 | 1 | .189 |
| The vector of trend weights is -3, -1, 1, 3. This is the default. | | | |

For T1-2 ESCC patients

| **Overall Comparisons** | | | |
| --- | --- | --- | --- |
|  | Chi-Square | df | Sig. |
| Log Rank (Mantel-Cox) | 14.968 | 1 | .000 |
| Breslow (Generalized Wilcoxon) | 16.044 | 1 | .000 |
| Tarone-Ware | 15.880 | 1 | .000 |
| The vector of trend weight is - 1, 0, 1. This is the default. | | | |

For T3-4 ESCC patients

| **Overall Comparisons** | | | |
| --- | --- | --- | --- |
|  | Chi-Square | df | Sig. |
| Log Rank (Mantel-Cox) | 13.749 | 1 | .000 |
| Breslow (Generalized Wilcoxon) | 16.377 | 1 | .000 |
| Tarone-Ware | 15.544 | 1 | .000 |
| The vector of trend weights is -3, -1, 1, 3. This is the default. | | | |

For ESCC patients with negative lymph node metastasis

| **Overall Comparisons** | | | |
| --- | --- | --- | --- |
|  | Chi-Square | df | Sig. |
| Log Rank (Mantel-Cox) | 7.976 | 1 | .005 |
| Breslow (Generalized Wilcoxon) | 10.217 | 1 | .001 |
| Tarone-Ware | 9.402 | 1 | .002 |
| The vector of trend weights is -3, -1, 1, 3. This is the default. | | | |

For ESCC patients with positive lymph node metastasis

| **Overall Comparisons** | | | |
| --- | --- | --- | --- |
|  | Chi-Square | df | Sig. |
| Log Rank (Mantel-Cox) | 1.658 | 1 | .198 |
| Breslow (Generalized Wilcoxon) | 1.648 | 1 | .199 |
| Tarone-Ware | 1.677 | 1 | .195 |
| The vector of trend weights is -3, -1, 1, 3. This is the default. | | | |

For all ESCC patients

| **Overall Comparisons** | | | |
| --- | --- | --- | --- |
|  | Chi-Square | df | Sig. |
| Log Rank (Mantel-Cox) | 19.430 | 1 | .000 |
| Breslow (Generalized Wilcoxon) | 20.010 | 1 | .000 |
| Tarone-Ware | 20.433 | 1 | .000 |
| The vector of trend weights is -3, -1, 1, 3. This is the default. | | | |

| **Means and Medians for Survival Time** | | | | | | | | |
| --- | --- | --- | --- | --- | --- | --- | --- | --- |
|  | Mean^a^ | | | | Median | | | |
|  | Estimate | Std. Error | 95% Confidence Interval | | Estimate | Std. Error | 95% Confidence Interval | |
|  |  |  | Lower Bound | Upper Bound |  |  | Lower Bound | Upper Bound |
| V0N0 | 67.052 | 4.494 | 58.243 | 75.860 | 90.000 | 21.293 | 48.266 | 131.734 |
| V1N0 | 34.292 | 4.153 | 26.152 | 42.432 | 21.000 | 3.937 | 13.283 | 28.717 |
| V0N1 | 48.000 | 12.920 | 22.678 | 73.322 | 23.000 | 10.435 | 2.547 | 43.453 |
| V1N1 | 32.384 | 5.152 | 22.286 | 42.482 | 20.000 | 4.594 | 10.997 | 29.003 |
| Overall | 51.726 | 3.193 | 45.468 | 57.984 | 36.000 | 5.614 | 24.996 | 47.004 |
| a. Estimation is limited to the largest survival time if it is censored. | | | | | | | | |

**3、Comparison of Kaplan–Meier curves stratified by whether postoperative adjuvant chemotherapy.**

For all ESCC patients with V0N0

| **Overall Comparisons** | | | |
| --- | --- | --- | --- |
|  | Chi-Square | df | Sig. |
| Log Rank (Mantel-Cox) | 2.575 | 1 | .109 |
| Breslow (Generalized Wilcoxon) | 3.011 | 1 | .083 |
| Tarone-Ware | 3.157 | 1 | .076 |
| The vector of trend weights is -1, 1. This is the default. | | | |

For all ESCC patients with V1N0

|  | | | |
| --- | --- | --- | --- |
|  | Chi-Square | df | Sig. |
| Log Rank (Mantel-Cox) | .007 | 1 | .933 |
| Breslow (Generalized Wilcoxon) | .492 | 1 | .483 |
| Tarone-Ware | .166 | 1 | .684 |
| The vector of trend weights is -1, 1. This is the default. | | | |

For all ESCC patients with V0N1

|  | | | |
| --- | --- | --- | --- |
|  | Chi-Square | df | Sig. |
| Log Rank (Mantel-Cox) | 2.047 | 1 | .153 |
| Breslow (Generalized Wilcoxon) | 1.602 | 1 | .206 |
| Tarone-Ware | 1.821 | 1 | .177 |
| The vector of trend weights is -1, 1. This is the default. | | | |

For all ESCC patients with V1N1

|  | | | |
| --- | --- | --- | --- |
|  | Chi-Square | df | Sig. |
| Log Rank (Mantel-Cox) | 8.457 | 1 | .004 |
| Breslow (Generalized Wilcoxon) | 6.382 | 1 | .012 |
| Tarone-Ware | 7.455 | 1 | .006 |
| The vector of trend weights is -1, 1. This is the default. | | | |

4、The Risk Factors associated with cancer specific survival using Chi square test.

**Age**

| **Age * category Crosstabulation** | | | | | |
| --- | --- | --- | --- | --- | --- |
|  | | | category | | Total |
|  |  |  | V0N0 | V1N1 |  |
| Age | < 60 | Count | 74 | 18 | 92 |
|  |  | Expected Count | 83.5 | 8.5 | 92.0 |
|  |  | % within Age | 80.4% | 19.6% | 100.0% |
|  | ≥ 60 | Count | 103 | 0 | 103 |
|  |  | Expected Count | 93.5 | 9.5 | 103.0 |
|  |  | % within Age | 100.0% | 0.0% | 100.0% |
| Total | | Count | 177 | 18 | 195 |
|  |  | Expected Count | 177.0 | 18.0 | 195.0 |
|  |  | % within Age | 90.8% | 9.2% | 100.0% |

| **Chi-Square Tests** | | | | |
| --- | --- | --- | --- | --- |
|  | Value | df | Asymptotic Significance (2-sided) | Exact Sig. (2-sided) |
| Pearson Chi-Square | 1.708^a^ | 2 | .426 | .451 |
| Likelihood Ratio | 1.724 | 2 | .422 | .451 |
| Fisher's Exact Test | 1.671 |  |  | .451 |
| N of Valid Cases | 195 |  |  |  |
| a. 2 cells (33.3%) have expected count less than 5. The minimum expected count is 4.25. | | | | |

**Sex**

| **Sex* category Crosstabulation** | | | | | |
| --- | --- | --- | --- | --- | --- |
|  | | | category | | Total |
|  |  |  | V0N0 | V1N1 |  |
| Sex | Male | Count | 111 | 29 | 140 |
|  |  | Expected Count | 116.3 | 23.7 | 140.0 |
|  |  | % within Sex | 79.3% | 20.7% | 100.0% |
|  | Female | Count | 51 | 4 | 55 |
|  |  | Expected Count | 45.7 | 9.3 | 55.0 |
|  |  | % within Sex | 92.7% | 7.3% | 100.0% |
| Total | | Count | 162 | 33 | 195 |
|  |  | Expected Count | 162.0 | 33.0 | 195.0 |
|  |  | % within Sex | 83.1% | 16.9% | 100.0% |

| **Chi-Square Tests** | | | | | |
| --- | --- | --- | --- | --- | --- |
|  | Value | df | Asymptotic Significance (2-sided) | Exact Sig. (2-sided) | Exact Sig. (1-sided) |
| Pearson Chi-Square | 5.075^a^ | 1 | .024 | .032 | .016 |
| Continuity Correction^b^ | 4.163 | 1 | .041 |  |  |
| Likelihood Ratio | 5.808 | 1 | .016 | .022 | .016 |
| Fisher's Exact Test |  |  |  | .032 | .016 |
| N of Valid Cases | 195 |  |  |  |  |
| a. 0 cells (0.0%) have expected count less than 5. The minimum expected count is 9.31. | | | | | |
| b. Computed only for a 2x2 table | | | | | |

**Location**

| **location * category Crosstabulation** | | | | | |
| --- | --- | --- | --- | --- | --- |
|  | | | category | | Total |
|  |  |  | V0N0 | V1N1 |  |
| location | Upper | Count | 25 | 3 | 28 |
|  |  | Expected Count | 23.3 | 4.7 | 28.0 |
|  |  | % within location | 89.3% | 10.7% | 100.0% |
|  | Lower | Count | 51 | 19 | 70 |
|  |  | Expected Count | 58.2 | 11.8 | 70.0 |
|  |  | % within location | 72.9% | 27.1% | 100.0% |
|  | Middle | Count | 86 | 11 | 97 |
|  |  | Expected Count | 80.6 | 16.4 | 97.0 |
|  |  | % within location | 88.7% | 11.3% | 100.0% |
| Total | | Count | 162 | 33 | 195 |
|  |  | Expected Count | 162.0 | 33.0 | 195.0 |
|  |  | % within location | 83.1% | 16.9% | 100.0% |

| **Chi-Square Tests** | | | | |
| --- | --- | --- | --- | --- |
|  | Value | df | Asymptotic Significance (2-sided) | Exact Sig. (2-sided) |
| Pearson Chi-Square | 8.118^a^ | 2 | .017 | .019 |
| Likelihood Ratio | 7.804 | 2 | .020 | .022 |
| Fisher's Exact Test | 7.501 |  |  | .025 |
| N of Valid Cases | 195 |  |  |  |
| a. 1 cells (16.7%) have expected count less than 5. The minimum expected count is 4.74. | | | | |

**Grade**

| **Grade * category Crosstabulation** | | | | | |
| --- | --- | --- | --- | --- | --- |
|  | | | category | | Total |
|  |  |  | V0N0 | V1N1 |  |
| Grade | Poor | Count | 28 | 10 | 38 |
|  |  | Expected Count | 31.6 | 6.4 | 38.0 |
|  |  | % within Grade | 73.7% | 26.3% | 100.0% |
|  | Well | Count | 28 | 8 | 36 |
|  |  | Expected Count | 29.9 | 6.1 | 36.0 |
|  |  | % within Grade | 77.8% | 22.2% | 100.0% |
|  | Moderate | Count | 106 | 15 | 121 |
|  |  | Expected Count | 100.5 | 20.5 | 121.0 |
|  |  | % within Grade | 87.6% | 12.4% | 100.0% |
| Total | | Count | 162 | 33 | 195 |
|  |  | Expected Count | 162.0 | 33.0 | 195.0 |
|  |  | % within 年龄 | 83.1% | 16.9% | 100.0% |

| **Chi-Square Tests** | | | | |
| --- | --- | --- | --- | --- |
|  | Value | df | Asymptotic Significance (2-sided) | Exact Sig. (2-sided) |
| Pearson Chi-Square | 4.867^a^ | 2 | .088 | .084 |
| Likelihood Ratio | 4.688 | 2 | .096 | .110 |
| Fisher's Exact Test | 5.004 |  |  | .079 |
| N of Valid Cases | 195 |  |  |  |
| a. 0 cells (0.0%) have expected count less than 5. The minimum expected count is 6.09. | | | | |

**T stage**

| **T stage * category Crosstabulation** | | | | | |
| --- | --- | --- | --- | --- | --- |
|  | | | category | | Total |
|  |  |  | V0N0 | V1N1 |  |
| T stage | T1-2 | Count | 70 | 3 | 73 |
|  |  | Expected Count | 60.6 | 12.4 | 73.0 |
|  |  | % within T stage | 95.9% | 4.1% | 100.0% |
|  | T3-4 | Count | 92 | 30 | 122 |
|  |  | Expected Count | 101.4 | 20.6 | 122.0 |
|  |  | % within T stage | 75.4% | 24.6% | 100.0% |
| Total | | Count | 162 | 33 | 195 |
|  |  | Expected Count | 162.0 | 33.0 | 195.0 |
|  |  | % within T stage | 83.1% | 16.9% | 100.0% |

| **Chi-Square Tests** | | | | | |
| --- | --- | --- | --- | --- | --- |
|  | Value | df | Asymptotic Significance (2-sided) | Exact Sig. (2-sided) | Exact Sig. (1-sided) |
| Pearson Chi-Square | 13.626^a^ | 1 | .000 | .000 | .000 |
| Continuity Correction^b^ | 12.208 | 1 | .000 |  |  |
| Likelihood Ratio | 16.193 | 1 | .000 | .000 | .000 |
| Fisher's Exact Test |  |  |  | .000 | .000 |
| N of Valid Cases | 195 |  |  |  |  |
| a. 0 cells (0.0%) have expected count less than 5. The minimum expected count is 12.35. | | | | | |
| b. Computed only for a 2x2 table | | | | | |

**Primary tumor length （mm）**

| **length * category Crosstabulation** | | | | | |
| --- | --- | --- | --- | --- | --- |
|  | | | category | | Total |
|  |  |  | V0N0 | V1N1 |  |
| length | < 36 | Count | 94 | 10 | 104 |
|  |  | Expected Count | 86.4 | 17.6 | 104.0 |
|  |  | % within 年龄 | 90.4% | 9.6% | 100.0% |
|  | ≥ 36 | Count | 68 | 23 | 91 |
|  |  | Expected Count | 75.6 | 15.4 | 91.0 |
|  |  | % within 年龄 | 74.7% | 25.3% | 100.0% |
| Total | | Count | 162 | 33 | 195 |
|  |  | Expected Count | 162.0 | 33.0 | 195.0 |
|  |  | % within 年龄 | 83.1% | 16.9% | 100.0% |

| **Chi-Square Tests** | | | | | |
| --- | --- | --- | --- | --- | --- |
|  | Value | df | Asymptotic Significance (2-sided) | Exact Sig. (2-sided) | Exact Sig. (1-sided) |
| Pearson Chi-Square | 8.465^a^ | 1 | .004 | .004 | .003 |
| Continuity Correction^b^ | 7.388 | 1 | .007 |  |  |
| Likelihood Ratio | 8.586 | 1 | .003 | .004 | .003 |
| Fisher's Exact Test |  |  |  | .004 | .003 |
| N of Valid Cases | 195 |  |  |  |  |
| a. 0 cells (0.0%) have expected count less than 5. The minimum expected count is 15.40. | | | | | |
| b. Computed only for a 2x2 table | | | | | |

**Lymphatic invasion**

| **Lymphatic invasion * category Crosstabulation** | | | | | |
| --- | --- | --- | --- | --- | --- |
|  | | | category | | Total |
|  |  |  | V0N0 | V1N1 |  |
| Lymphatic invasion | No | Count | 86 | 5 | 91 |
|  |  | Expected Count | 75.6 | 15.4 | 91.0 |
|  |  | % within Lymphatic invasion | 94.5% | 5.5% | 100.0% |
|  | Yes | Count | 76 | 28 | 104 |
|  |  | Expected Count | 86.4 | 17.6 | 104.0 |
|  |  | % within Lymphatic invasion | 73.1% | 26.9% | 100.0% |
| Total | | Count | 162 | 33 | 195 |
|  |  | Expected Count | 162.0 | 33.0 | 195.0 |
|  |  | % within Lymphatic invasion | 83.1% | 16.9% | 100.0% |

| **Chi-Square Tests** | | | | | |
| --- | --- | --- | --- | --- | --- |
|  | Value | df | Asymptotic Significance (2-sided) | Exact Sig. (2-sided) | Exact Sig. (1-sided) |
| Pearson Chi-Square | 15.851^a^ | 1 | .000 | .000 | .000 |
| Continuity Correction^b^ | 14.364 | 1 | .000 |  |  |
| Likelihood Ratio | 17.426 | 1 | .000 | .000 | .000 |
| Fisher's Exact Test |  |  |  | .000 | .000 |
| N of Valid Cases | 195 |  |  |  |  |
| a. 0 cells (0.0%) have expected count less than 5. The minimum expected count is 15.40. | | | | | |
| b. Computed only for a 2x2 table | | | | | |

**Stage**

| **Stage * category Crosstabulation** | | | | | |
| --- | --- | --- | --- | --- | --- |
|  | | | category | | Total |
|  |  |  | V0N0 | V1N1 |  |
| Stage | I-II | Count | 91 | 6 | 97 |
|  |  | Expected Count | 80.6 | 16.4 | 97.0 |
|  |  | % within Stage | 93.8% | 6.2% | 100.0% |
|  | III-IV | Count | 71 | 27 | 98 |
|  |  | Expected Count | 81.4 | 16.6 | 98.0 |
|  |  | % within Stage | 72.4% | 27.6% | 100.0% |
| Total | | Count | 162 | 33 | 195 |
|  |  | Expected Count | 162.0 | 33.0 | 195.0 |
|  |  | % within Stage | 83.1% | 16.9% | 100.0% |

| **Chi-Square Tests** | | | | | |
| --- | --- | --- | --- | --- | --- |
|  | Value | df | Asymptotic Significance (2-sided) | Exact Sig. (2-sided) | Exact Sig. (1-sided) |
| Pearson Chi-Square | 15.828^a^ | 1 | .000 | .000 | .000 |
| Continuity Correction^b^ | 14.345 | 1 | .000 |  |  |
| Likelihood Ratio | 16.925 | 1 | .000 | .000 | .000 |
| Fisher's Exact Test |  |  |  | .000 | .000 |
| N of Valid Cases | 195 |  |  |  |  |
| a. 0 cells (0.0%) have expected count less than 5. The minimum expected count is 16.42. | | | | | |
| b. Computed only for a 2x2 table | | | | | |

5、The Risk Factors associated with cancer specific survival using univariate logistic regression analysis.

**Age**

| **Variables in the Equation** | | | | | | | | | |
| --- | --- | --- | --- | --- | --- | --- | --- | --- | --- |
|  | | B | S.E. | Wald | df | Sig. | Exp(B) | 95% C.I.for EXP(B) | |
|  |  |  |  |  |  |  |  | Lower | Upper |
| Step 1^a^ | ≥ 60 | -.356 | .384 | .860 | 1 | .354 | .701 | .330 | 1.486 |
|  | Constant | -1.414 | .263 | 28.935 | 1 | .000 | .243 |  |  |
| a. Variable(s) entered on step 1: Age. | | | | | | | | | |

**Sex**

| **Variables in the Equation** | | | | | | | | | |
| --- | --- | --- | --- | --- | --- | --- | --- | --- | --- |
|  | | B | S.E. | Wald | df | Sig. | Exp(B) | 95% C.I.for EXP(B) | |
|  |  |  |  |  |  |  |  | Lower | Upper |
| Step 1^a^ | Female | -1.203 | .560 | 4.624 | 1 | .032 | .300 | .100 | .899 |
|  | Constant | -1.342 | .209 | 41.424 | 1 | .000 | .261 |  |  |
| a. Variable(s) entered on step 1: Sex. | | | | | | | | | |

**Tumor location**

| **Variables in the Equation** | | | | | | | | | |
| --- | --- | --- | --- | --- | --- | --- | --- | --- | --- |
|  | | B | S.E. | Wald | df | Sig. | Exp(B) | 95% C.I.for EXP(B) | |
|  |  |  |  |  |  |  |  | Lower | Upper |
| Step 1^a^ | location |  |  | 7.688 | 2 | .021 |  |  |  |
|  | Lower | 1.133 | .668 | 2.880 | 1 | .090 | 3.105 | .839 | 11.487 |
|  | Middle | .064 | .690 | .009 | 1 | .926 | 1.066 | .276 | 4.120 |
|  | Constant | -2.120 | .611 | 12.042 | 1 | .001 | .120 |  |  |
| a. Variable(s) entered on step 1: location. | | | | | | | | | |

**Lymphatic invasion**

| **Variables in the Equation** | | | | | | | | | |
| --- | --- | --- | --- | --- | --- | --- | --- | --- | --- |
|  | | B | S.E. | Wald | df | Sig. | Exp(B) | 95% C.I.for EXP(B) | |
|  |  |  |  |  |  |  |  | Lower | Upper |
| Step 1^a^ | Lymphatic invasion (+) | 1.846 | .510 | 13.087 | 1 | .000 | 6.337 | 2.330 | 17.231 |
|  | Constant | -2.845 | .460 | 38.244 | 1 | .000 | .058 |  |  |
| a. Variable(s) entered on step 1: N. | | | | | | | | | |

**Stage**

| **Variables in the Equation** | | | | | | | | | |
| --- | --- | --- | --- | --- | --- | --- | --- | --- | --- |
|  | | B | S.E. | Wald | df | Sig. | Exp(B) | 95% C.I.for EXP(B) | |
|  |  |  |  |  |  |  |  | Lower | Upper |
| Step 1^a^ | III-IV stage | 1.752 | .478 | 13.421 | 1 | .000 | 5.768 | 2.259 | 14.728 |
|  | Constant | -2.719 | .421 | 41.617 | 1 | .000 | .066 |  |  |
| a. Variable(s) entered on step 1: Stage. | | | | | | | | | |

**Primary tumor length （mm**）

| **Variables in the Equation** | | | | | | | | | |
| --- | --- | --- | --- | --- | --- | --- | --- | --- | --- |
|  | | B | S.E. | Wald | df | Sig. | Exp(B) | 95% C.I.for EXP(B) | |
|  |  |  |  |  |  |  |  | Lower | Upper |
| Step 1^a^ | ≥ 36 | 1.157 | .411 | 7.925 | 1 | .005 | 3.179 | 1.421 | 7.114 |
|  | Constant | -2.241 | .333 | 45.380 | 1 | .000 | .106 |  |  |
| a. Variable(s) entered on step 1: length. | | | | | | | | | |

**Tumor types**

| **Variables in the Equation** | | | | | | | | | |
| --- | --- | --- | --- | --- | --- | --- | --- | --- | --- |
|  | | B | S.E. | Wald | df | Sig. | Exp(B) | 95% C.I.for EXP(B) | |
|  |  |  |  |  |  |  |  | Lower | Upper |
| Step 1^a^ | Tumor types |  |  | 10.926 | 2 | .004 |  |  |  |
|  | Ulcerative | 2.028 | .649 | 9.773 | 1 | .002 | 7.600 | 2.131 | 27.104 |
|  | Medullary | 1.240 | .685 | 3.276 | 1 | .070 | 3.455 | .902 | 13.224 |
|  | Constant | -2.944 | .592 | 24.709 | 1 | .000 | .053 |  |  |
| a. Variable(s) entered on step 1: Tumor types. | | | | | | | | | |

5、The Risk Factors associated with cancer specific survival using multivariate logistic regression analysis.

| **Variables in the Equation** | | | | | | | | | |
| --- | --- | --- | --- | --- | --- | --- | --- | --- | --- |
|  | | B | S.E. | Wald | df | Sig. | Exp(B) | 95% C.I.for EXP(B) | |
|  |  |  |  |  |  |  |  | Lower | Upper |
| Step | Female | -1.061 | .602 | 3.110 | 1 | .078 | .346 | .106 | 1.125 |
|  | T3-4 | 1.518 | .801 | 3.595 | 1 | .058 | 4.565 | .950 | 21.930 |
|  | Lymphatic invasion | 2.394 | 1.233 | 3.769 | 1 | .052 | 10.960 | .977 | 122.917 |
|  | III-IV stage | -1.030 | 1.222 | .711 | 1 | .399 | .357 | .033 | 3.914 |
|  | Tumor types |  |  | 5.190 | 2 | .075 |  |  |  |
|  | Ulcerative | 1.459 | .711 | 4.210 | 1 | .040 | 4.302 | 1.068 | 17.332 |
|  | Medullary | .776 | .751 | 1.068 | 1 | .301 | 2.172 | .499 | 9.462 |
|  | ≥ 36 | .677 | .451 | 2.257 | 1 | .133 | 1.968 | .814 | 4.762 |
|  | Constant | -4.877 | 1.017 | 22.972 | 1 | .000 | .008 |  |  |
|  | | | | | | | | | |
